# War Shock. The Psychoneuroses in War Psychology and Treatment

**Published:** 1919

**Authors:** 


### Reviews of Books.

**War Shock. The Psychoneuroses in War Psychology and Treatment.** By M. D. Eder, B.Sc. Lond., M.R.C.S., L.R.C.P. Lond. Pp. 154. London: William Heinemann. (n.d.) Price 5s. net.

The writer is the authorised translator of Freud's book on "Dreams," and he has taken for analysis the first hundred cases of psychoneurosis which came under his care to show how well soldiers suffering from war shock respond to mental treatment, claiming that most shell shock cases can be relieved in a few days. He holds that war shock is hysteria in healthy persons who are specially responsive to psychical stimuli when exposed to the strain of war conditions. Their symptoms are due to mental conflicts. Neuropathic predisposition has little to do with it in the majority of cases. The best treatment is hypnotic suggestion after a psycho-analytical examination. Other forms of suggestion are less rapid and more uncertain. Most cases are well in less than two weeks. Of the true cases, 91.5 per cent. were cured and 8.5 per cent. improved, but in those where neuropathic antecedents existed 62 per cent. only were cured and 27.6 per cent. improved. His description of the clinical symptoms and their explanation is remarkably interesting and valuable; the amblyopias, the left-sided hemianæsthesias, the irritable heart, aphonia, paralyses and spasms, have been carefully studied, though we fear that he is more hopeful of cure than our present knowledge will justify.

